# Preliminary Study on the Efficacy of a Recombinant, Subunit SARS-CoV-2 Animal Vaccine against Virulent SARS-CoV-2 Challenge in Cats

**DOI:** 10.3390/vaccines11121831

**Published:** 2023-12-08

**Authors:** Igor Morozov, Natasha N. Gaudreault, Jessie D. Trujillo, Sabarish V. Indran, Konner Cool, Taeyong Kwon, David A. Meekins, Velmurugan Balaraman, Bianca Libanori Artiaga, Daniel W. Madden, Chester McDowell, Bradley Njaa, Jamie Retallick, Nicole Hainer, Jason Millership, William C. Wilson, George Tkalcevic, Hanne Vander Horst, Yulia Burakova, Vickie King, Kendra Hutchinson, John M. Hardham, Denise J. Schwahn, Mahesh Kumar, Juergen A. Richt

**Affiliations:** 1 Department of Diagnostic Medicine/Pathobiology, College of Veterinary Medicine, Kansas State University, Manhattan, KS 66506, USAbalarama@vet.k-state.edu (V.B.);; 2 Kansas State Veterinary Diagnostic Laboratory, Kansas State University, Manhattan, KS 66506, USA; blnjaa@vet.k-state.edu (B.N.);; 3 Zoetis, Kalamazoo, MI 49007, USA; 4 Foreign Arthropod-Borne Animal Disease Research Unit, National Bio and Agro-Defense Facility, United States Department of Agriculture, Manhattan, KS 66506, USA

**Keywords:** SARS-CoV-2, cats, feline, recombinant vaccine, virus-neutralizing antibodies, immune response

## Abstract

The objective of this work was to evaluate the safety and efficacy of a recombinant, subunit SARS-CoV-2 animal vaccine in cats against virulent SARS-CoV-2 challenge. Two groups of cats were immunized with two doses of either a recombinant SARS-CoV-2 spike protein vaccine or a placebo, administered three weeks apart. Seven weeks after the second vaccination, both groups of cats were challenged with SARS-CoV-2 via the intranasal and oral routes simultaneously. Animals were monitored for 14 days post-infection for clinical signs and viral shedding before being humanely euthanized and evaluated for macroscopic and microscopic lesions. The recombinant SARS-CoV-2 spike protein subunit vaccine induced strong serologic responses post-vaccination and significantly increased neutralizing antibody responses post-challenge. A significant difference in nasal and oral viral shedding, with significantly reduced virus load (detected using RT-qPCR) was observed in vaccinates compared to mock-vaccinated controls. Duration of nasal, oral, and rectal viral shedding was also significantly reduced in vaccinates compared to controls. No differences in histopathological lesion scores were noted between the two groups. Our findings support the safety and efficacy of the recombinant spike protein-based SARS-CoV-2 vaccine which induced high levels of neutralizing antibodies and reduced nasal, oral, and rectal viral shedding, indicating that this vaccine will be efficacious as a COVID-19 vaccine for domestic cats.

## 1. Introduction

Since the beginning of the SARS-CoV-2 pandemic, the virus has continued to spread and circulate throughout the human population on a global scale. Along with humans, multiple animal species have been shown to be susceptible to SARS-CoV-2 infection; this could play an important role in the ecology of SARS-CoV-2 and might contribute to the evolution of the virus via dissemination and persistence in susceptible animal species [1,2]. Highly susceptible species include primates, domestic and large cats, dogs, ferrets, mink, hamsters, and white-tailed deer, as well as multiple other animal species with varying degrees of susceptibility [3,4,5,6,7,8,9,10,11,12,13,14,15]. The emergence of new viral variants with spike mutations that broaden host susceptibility has already been demonstrated in mice [16].

Domestic and large cats are highly susceptible species to SARS-CoV-2 infection as demonstrated by natural infections reported worldwide [17,18,19,20,21]. There are multiple reports on the seroprevalence of SARS-CoV-2 among domestic cats and cases of virus transmission from infected owners to their pet felines [22,23,24,25]. Recently, researchers in Thailand reported the first documented case of SARS-CoV-2 spillover from cats to humans [26]. Several reports describe the incidence of SARS-CoV-2 infection among large cats kept in zoos around the world. SARS-CoV-2 was detected in lions at the Barcelona zoo [22,27], in lions and tigers at the Bronx zoo in New York [28], snow leopards at a zoo in Kentucky [29], and pumas and lions at a zoo in South Africa [30]. A case of lion-to-human transmission was recently reported in a zoo while an infected lion was undergoing treatment [31]. Multiple reports on experimental infection of cats demonstrate productive infection and efficient transmission to sentinel animals [32,33,34,35]. All this demonstrates that feline species are highly susceptible to SARS-CoV-2 infection and may play a role in the epidemiology of SARS-CoV-2.

Considering the large number of pet cats in households as well as feral cats in the US and abroad, there is concern that cats may contribute to the maintenance and spread of SARS-CoV-2, provide a virus reservoir, and may contribute to the emergence of new virus variants [36,37]. Thus, it is important to develop effective means for the control of SARS-CoV-2 infection in cats. Vaccines are an important tool for the control of SARS-CoV-2. Multiple types of vaccines have been developed and are used in humans worldwide. These include mRNA vaccines, vector-based vaccines, inactivated vaccines, and recombinant subunit vaccines [38,39,40]. To address concerns about SARS-CoV-2 infection and disease in domestic animals, Zoetis developed a recombinant subunit vaccine based on the eukaryotic cell-expressed spike protein of SARS-CoV-2. This experimental vaccine was developed for mink and was also authorized for experimental use on a case-by-case basis in zoos, conservatories, and aquariums of several animal organizations in the USA and other countries to vaccinate various mammalian species, including tigers, mountain lions, primates, and other species (https://news.zoetis.com/press-releases/press-release-details/2021/Zoetis-Donates-COVID-19-Vaccines-to-Help-Support-the-Health-of-Zoo-Animals/default.aspx, accessed on 6 June 2023).

In the present study, the safety and efficacy of the Zoetis SARS-CoV-2 vaccine was evaluated in a two-dose vaccination regime against virulent SARS-CoV-2 challenge in domestic cats. We report here that the vaccine induces high titers of neutralizing and protective antibody responses in cats, and significantly reduced viral shedding in vaccinates compared to mock controls following SARS-CoV-2 challenge. The lack of clinical signs following vaccination supports the safety of the vaccine.

## 2. Materials and Methods

### 2.1. Study Design

Cats were sourced from Marshall BioResources Specific Pathogen Free cat colony. In this facility, cats are raised unvaccinated and free of a variety of feline pathogens such as feline herpesvirus, feline calicivirus, feline panleukopenia, and feline coronavirus. The colony is historically negative for FIV, feline chlamydia, and toxoplasmosis. Of the cats enrolled in this study, half in each treatment group were previously vaccinated at 8–10 week of age with 2 doses of an experimental feline leukemia virus (FeLV) vaccine given 3 weeks apart. The other half of the cats were exposed to a FeLV challenge only. All cats were FeLV negative by serology when enrolled in the present vaccination study. The study included a total of 5 castrated males and 3 intact females.

In the present study, two treatment groups, T01 and T02, with four cats per treatment group were either mock-immunized (placebo) or immunized with the Zoetis COVID-19 vaccine and after the booster vaccination transported to the Kansas State University’s high-containment Biosecurity Research Institute (BRI). Treatment groups T01 (placebo group) and T02 (COVID-19 vaccine group) were treated with two subcutaneous doses of placebo or COVID-19 vaccine, respectively, administered three weeks apart. The vaccine comprised a stabilized, recombinant SARS-CoV-2 spike ectodomain based on the Wuhan-1 SARS-CoV-2 strain formulated with a proprietary adjuvant system. The vaccination phase was performed at Zoetis, and the challenge phase of the study was performed at the BRI at Kansas State University. The animals were randomly selected for inclusion in the T01 and T02 groups using the SAS program with a random number generator. Cats were 8 to 11 months of age at the time of first vaccine dose, and the SARS-CoV-2 challenge was performed seven weeks after the booster vaccination, i.e., they were approximately 10–14 months old at the time of SARS-CoV-2 challenge. Post-challenge, animals were observed for 14 days for clinical signs. Nasal, oral, and rectal swabs were collected to monitor viral shedding, and blood samples were collected to monitor post-challenge immune responses. On day 14 post-challenge, all animals in the study were humanely euthanized and necropsied. At necropsy, gross pathologic observations were performed, and tissues were collected for microscopic examination. This research project was reviewed and approved by IBC and IACCUC committees for compliance with Kansas State University research protocols for biomedical and animal research.

### 2.2. Cells, Virus, and Virus Challenge Procedure

The SARS-CoV-2 USA-WA1/2020 strain was used for the cat challenge. At the time this study was conducted, this was the relevant SARS-CoV-2 strain and it was also the strain used to establish the cat COVID-19 model in our laboratory. Furthermore, the vaccine in this study was produced based on the sequence of the Wuhan prototype strain; therefore, a similar strain was used for homologous challenge to demonstrate protective properties of the vaccine. The SARS-CoV-2 USA-WA1/2020 virus was acquired from the Biodefense and Emerging Infection (BEI) Research Resources Repository (catalog # NR-52281, BEI Resources, Manassas, VA, USA). The virus was passaged 3 times in Vero E6 cells to establish a stock virus for inoculation of animals. This stock virus was sequenced using next-generation sequencing (NGS) using the Illumina MiSeq and its consensus sequence was found to be 100% homologous to the original USA-WA1/2020 strain (GenBank accession: MN985325.1). The SARS-CoV-2 stock was diluted in DMEM for a final concentration of 5 × 10^5^ TCID_50_/mL; this virus concentration was used for experimental infection of the cats. For infection of cats, 2 mL of inoculum was administered: 0.5 mL per nostril and 1 mL orally for a 1 × 10^6^ TCID_50_ total dose per cat. A back titration, performed from an aliquot of diluted challenge material, confirmed the intended dose of challenge material.

### 2.3. Clinical Observations and Sample Collection

All animals were observed daily, starting on days −2, −1, and 0 before/on the day of challenge, and then daily until study completion (day 14 post-infection); they were observed for clinical signs of fever, conjunctivitis, depression, dehydration, nasal congestion, nasal discharge, cough, ocular discharge, sneezing, or other abnormalities. Nasal, oral, and rectal swabs were collected before the challenge on day 0 and on days 2, 4, 6, 8, 10, 12, and 14 post-challenge. Swabs were placed in 2 mL virus transport medium (VTM; DMEM; Corning, New York, NY, USA) with addition of antibiotics/antimycotic (Fisher Scientific, Waltham, MA, USA). In the laboratory, swabs were vortexed and the supernatant was aliquoted directly into cryovials with RLT buffer (Qiagen, Germantown, MD, USA), and stored at −80 °C until further analysis. Blood samples for serology (1–2 mL) were collected from all animals on day 0 and at necropsy on day 14.

### 2.4. Virus-Neutralizing Antibodies Test

Virus neutralizing antibodies in sera were determined using a microneutralization assay. Briefly, serum samples were initially diluted at 1:10 and heat-inactivated (56 °C for 30 min while shaking). Subsequently, 100 μL of serum per well in duplicates was subjected to 2-fold serial dilutions (1:20 through 1:2560) in 100 μL culture media. Then, 100 μL of 100 TCID_50_ SARS-CoV-2 virus (strain USA-WA1/2020) in culture media was added to 100 μL of the sera dilutions, and incubated for 1 h at 37 °C. The 200 μL of virus–serum mixture was then applied on Vero E6 cells in 96-well plates. The corresponding SARS-CoV-2 negative and positive cat sera, virus-only and media-only controls were included in the assay. Results of the virus neutralization test were determined by endpoint titration and calculated as 50% of neutralization (NT_50_) based on the appearance of CPE observed under a microscope at 72 h post-infection.

### 2.5. ELISA Antibody Test

Sera were tested using an *in-house* indirect ELISA based on the recombinant RBD protein of Wuhan-like SARS-CoV-2 (developed by Zoetis). For the indirect ELISA, wells were coated with 100 ng of the RBD protein in 100 μL coating buffer (carbonate–bicarbonate buffer, #C3041, Sigma-Aldrich, St. Louis, MO, USA) per well and incubated overnight at 4 °C. The next day, the plates were washed 2 times with PBS (phosphate-buffered saline, #P4417, Sigma-Aldrich, St. Louis, MO, USA), blocked with 200 μL casein blocking buffer (Sigma-Aldrich, #B6429, St. Louis, MO, USA) per well, and incubated for 1 h at room temperature before being washed 3 times with PBS–Tween20 (PBS-T; 0.5% Tween20). Serum samples were pre-diluted at 1:400 in casein blocking buffer, then 100 μL pre-diluted serum was added to the ELISA plate wells and incubated for 1 h at room temperature. The wells were then washed 3 times with PBS-T, and 100 μL of goat anti-feline IgG (H+L) secondary antibody, HRP (ThermoFisher Scientific, #A18757, Waltham, MA, USA), diluted 1:2500 in PBS, was added to each well and incubated for 1 h at room temperature. After 1 h, the plate was washed 5 times with PBS-T, and 100 μL of TMB ELISA substrate solution (Abcam, #ab171525, Cambridge, MA, USA) was added to all wells of the plate. Following incubation at room temperature for 5 min, the reaction was stopped by adding 100 μL of stop solution for TMB Substrate (Abcam, #ab171529, Cambridge, MA, USA) to all wells. The ODs of the ELISA plates were read at 450 nm on an ELx808 BioTek plate reader (BioTek, Winooski, VT, USA). The cut-off for a sample being called positive was determined as an average OD of negative sera + 3X standard deviation. Everything above the cut-off value was considered positive.

### 2.6. RT-qPCR for Detection of SARS-CoV-2 in Tissues and Nasal, Oral, and Rectal Swabs

RNA was extracted from 100 μL of samples in virus transport media (VTM) by combining the sample with an equal amount of RLT RNA stabilization/lysis buffer (Qiagen, Germantown, MD, USA), followed by extraction using a magnetic bead-based nucleic acid extraction kit (GeneReach USA, Lexington, MA, USA) on an automated Taco^TM^ mini nucleic acid extraction system (GeneReach USA) according to the manufacturer’s protocol. Quantification of SARS-CoV-2 RNA was performed using the N2 SARS-CoV-2 primer and probe sets (see: https://www.idtdna.com/pages/landing/coronavirus-research-reagents/cdc-assays, accessed on 31 March 2020) using an RT-qPCR protocol established by the CDC for the detection of SARS-CoV-2 nucleoprotein (N)-specific RNA (https://www.fda.gov/media/134922/download, accessed on 17 March 2020). Data are expressed as the mean of the calculated N gene copy number per 1 mL of liquid sample or per mg of a 20% tissue homogenate.

### 2.7. Necropsy Procedures and Microscopic Examinations (Histopathology)

Following humane euthanasia, tissue samples from the respiratory tract (nasal turbinates, trachea/bronchi, and lungs), liver, heart, spleen, and kidney were collected and fixed in 10% neutral-buffered formalin for histopathologic evaluation. In addition, nasal turbinates, trachea/bronchi, and lungs were collected and frozen at −80 °C for virological assays. Formalin-fixed tissues were routinely trimmed, processed, embedded, sectioned, and stained with hematoxylin and eosin (H&E) following standard procedures. Upper and lower airways (nasal turbinates, trachea, and bronchi) were evaluated for changes in the lining epithelium, submucosal glands and ducts, and inflammatory cell infiltration. The pulmonary parenchyma was examined for changes in alveolar spaces and alveolar septa (e.g., inflammatory cell infiltration and exudation); intrapulmonary bronchi and bronchioles (e.g., alterations in the lining epithelium and submucosal glands, intraluminal exudate), and pulmonary vessels (e.g., perivascular cuffing and possible fibrin thrombi) were also analyzed. Two independent veterinary pathologists examined the slides blinded and recorded their evaluations. Histopathological abnormalities were scored for severity (0, not present; 1, minimal; 2, mild; 3, moderate; 4, marked; 5, severe). A third, blinded pathologist provided possible diagnoses for each tissue examined, reviewed the data, and resolved conflicts in severity scores and diagnostic terminology to provide consensus data, after analyzing the data unblinded.

### 2.8. Statistical Analyses

The primary efficacy variable was virus RNA detection via RT-qPCR. Hypothesis tests were conducted at the 0.10 level of significance (two-tailed). Antibody titers, clinical signs, and gross organ lesions +/− histopathologic evidence were treated as supporting data. Antibody data were logarithmically transformed before analysis with a general linear mixed model for repeated measurements. The fixed effects in the model were treatment, time point, and treatment by time point interaction. The random effects in the model were the block, the animal within the block and treatment, and error. The treatment groups were compared at each time point using contrasts. Treatment least squares mean, standard errors, and 90% confidence limits were back-transformed for each time point. Treatment minimums and maximums for each time point were also calculated.

Nasal, oral, and rectal swab RT-qPCR data were analyzed using the same method as described above for the antibody data. Duration of virus RNA detected using RT-qPCR in nasal, oral and rectal swabs were calculated as ((last day detected − first day detected) +1) for animals with positive results and set to 0 for animals with no virus RNA detected. Area under the curve (AUC) of RT-qPCR data from nasal, oral, and rectal swabs was calculated for each animal using the trapezoidal rule. Durations of virus RNA detected and logarithm-transformed AUCs were analyzed with a general linear mixed model with the fixed effect of treatment and the random effects of block and error. Treatment least squares means, standard errors, and 90% confidence limits were calculated and back-transformed if transformed for analysis. Treatment minimums and maximums were also calculated.

## 3. Results

### 3.1. Clinical Observations: Vaccination Phase

There were no immediate reactions, palpable injection site reactions, or abnormal clinical signs observed post-vaccination. A slight fever was observed in two animals of the COVID-19 vaccine group T02 (39.6–39.8 °C), before resolving the next day.

### 3.2. Clinical Observations: SARS-CoV-2 Challenge Phase

The only abnormal clinical observation identified during the SARS-CoV-2 challenge phase was fever (≥103.0 °F), which was noted in one animal in the unvaccinated placebo group T01. This animal had episodic fever starting on day 2 and ending on day 10 (ranging from 103.2 °F to 103.5 °F). No animals in the COVID-19 vaccine group T02 had a fever or any other clinical observation at any time.

### 3.3. Determination of Serological Responses to Vaccination and Challenge

Two serological methods were used to identify and quantify the serological responses to vaccination and challenge.

#### 3.3.1. ELISA Antibody Test

All animals in the placebo control group T01 were negative (BT LSM ≤ 300) for antibody titers using the *in-house* ELISA based on the RBD protein on day 0 before the challenge, whereas the T02 vaccinated group had a BT LSM of 42089, indicative of a response to vaccination. On day 14 post-challenge, all animals had increased antibody titers due to SARS-CoV-2 challenge, with a BT LSM of 3553 for the T01 placebo control group and a BT LSM 95,865 for the T02 COVID-19 vaccine group (Table 1). A significant difference was observed between treatment groups on days 0 and 14 (*p* < 0.0001).

#### 3.3.2. Virus-Neutralizing Antibodies Test

Serum neutralization (SN) against the Wuhan-like SARS-CoV-2 strain USA-WA1/2020 was tested on day 0 and 14 post-challenge. All animals in the placebo control group T01 had negative SNs on day 0, whereas the vaccinated group T02 had a back-transformed least square mean (BT LSM) neutralizing antibody titer of 1:320, indicative of a good response to the SARS-CoV-2 spike protein subunit vaccine. On day 14 post-challenge, neutralizing antibody titers were 1:160 (BT LSM) for the T01 placebo control group and ≥1:2560 (BT LSM) for the T02 vaccinated group, the latter representing a serum dilution which was not end-pointed This indicates an anamnestic response in the vaccinated animals to SARS-CoV-2 challenge. The SN antibody titers BT LMSs for each treatment group are shown in Table 2. The SN antibody titer comparison between the two treatment groups demonstrated a significant difference in antibody titers when comparing the T02 vaccinated group to the T01 placebo control group on days 0 and 14 (*p* < 0.0001).

### 3.4. RT-qPCR Detection of SARS-CoV-2 in Tissues, and Nasal, Oral, and Rectal Swabs

The nasal, oral, and rectal swab RT-qPCR test results are summarized in Table 3. The T02vaccinated group showed significantly less nasal shedding when compared to the T01 control group on days 2-14 (*p* ≤ 0.0047). The back-transformed least square mean for the area under the curve (AUC) was 6.9 × 10^10^ for the T01 control animals and 2.3 × 10^7^ for the T02 vaccinates for nasal shedding (*p* = 0.01274). The vaccinated T02 group had decreased duration of nasal shedding of 7.5 days vs. 13.0 days in the T01 controls (*p* = 0.0060).

The T02 vaccinated group showed significantly less oral shedding on days 2, 8, and 14 when compared to the T01 control group (*p* ≤ 0.0711). The back-transformed least square mean for AUC was 4.87 × 10^9^ for the T01 control animals and 1.25 × 10^8^ for the T02 vaccinates for oral shedding (*p* = 0.01471). Vaccinated animals had decreased duration of oral shedding of 7.5 days vs. 10.0 days in the T01 controls (*p* = 0.0587).

Furthermore, the T02 vaccinated group showed significantly less rectal shedding on day 2 compared to the T01 control group (*p* < 0.0001). The back-transformed least square mean for AUC was 1.47 × 10^12^ for the T01 control animals and 9.80 × 10^9^ for the T02 vaccinates for rectal shedding (*p* = 0.21034). Vaccinated animals had decreased duration of rectal shedding of 7.5 days vs. 12.0 days in the T01 controls (*p* = 0.0547).

One animal in the T01 control group tested positive for viral RNA in the trachea, but negative in the nasal turbinates and lungs. Virus was not detected in the trachea, nasal turbinates, or lungs of any other animal.

### 3.5. Macroscopic and Microscopic Pathology

Macroscopic observations occurred at similar incidence and severity in both treatment groups and included congestion, discoloration, and atelectasis of the lungs. Two of four (50%) T01 control animals and two of four (50%) T02 vaccinated animals had lung atelectasis, and all of the animals in both groups had lung congestion and discoloration. Neither group had apparent lung edema or pneumonia. One cat in the T02 group had a 1.5 cm diameter hemorrhagic spot in the right ventricular wall of the heart. There were no other macroscopic findings.

**Figure 1:**
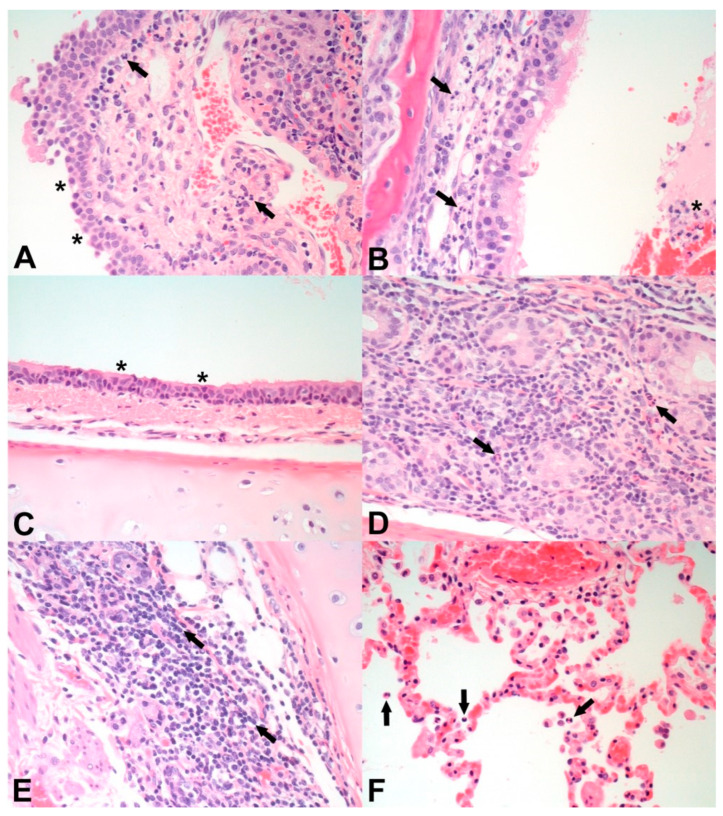
Representative photomicrographs of histopathological changes on day 14 post-challenge as observed in the nasal turbinates (**A**,**B**), trachea (**C**,**D**), bronchus (**E**), and lung (**F**) of cats infected with SARS-CoV-2 following mock vaccination (**A**,**C**,**E**) or SARS-CoV-2 vaccination (**B**,**D**,**F**). (**A**) Nasal turbinates of an unvaccinated, challenged cat (ID M192790) with epithelial attenuation (asterisks) and neutrophilic and lymphoplasmacytic inflammation in the lamina propria (arrows). (**B**) Nasal turbinates of a vaccinated, challenged cat (ID M191806) showing neutrophilic and lymphoplasmacytic inflammation in the lamina propria (arrows) and lumen (asterisk). (**C**) Trachea from an unvaccinated, challenged cat (ID M192790) with epithelial attenuation and segmental loss of cilia (asterisks). (**D**) Trachea from a vaccinated, challenged cat (ID M192812) exhibiting neutrophilic and lymphoplasmacytic inflammation around tracheal glands (arrows). (**E**) Bronchus from an unvaccinated, challenged cat (ID M192790) showing lymphoplasmacytic inflammation (arrows) and loss of bronchial glands. (**F**) Lung from a vaccinated, challenged cat (ID M192812) showing neutrophilic exudation into the alveoli (arrows) and congestion. Original magnification of all images: 400×.

Microscopic observations in the nasal turbinates, trachea/bronchi, and lungs were acute to subacute and attributed to the recent SARS-CoV-2 challenge. There were no chronic lesions suggestive of a reaction to vaccination in either group. Histopathologic changes in the nasal turbinates (Table 4) included mild epithelial attenuation with cilia loss (Figure 1A), minimal to moderate lymphoplasmacytic nasal gland adenitis, and minimal to marked lymphoplasmacytic rhinitis (Figure 1A,B) as shown in Figure 1 for animals in both treatment groups. The incidence and severity of lymphoplasmacytic adenitis and rhinitis were similar for both groups.

Microscopic observations in the trachea/bronchi (Table 4) included minimal to mild epithelial attenuation with cilia loss (Figure 1C) and minimal to moderate lymphoplasmacytic tracheal gland adenitis (Figure 1D) as shown in Figure 1 for animals in both treatment groups. The incidence and severity of lymphoplasmacytic tracheal gland adenitis were similar in both groups. Additionally, one cat in the T02 vaccinated group had minimal lymphoplasmacytic tracheitis.

Microscopic observations in the lung (Table 4) included minimal to mild exudative alveolitis (Figure 1F), minimal to moderate lymphoplasmacytic and eosinophilic bronchial adenitis (Figure 1E), minimal to mild lymphoplasmacytic perivascular cuffing, moderate to marked pulmonary congestion (correlating with the congestion and discoloration seen macroscopically; Figure 1F), and mild pulmonary edema as shown in Figure 1 for animals in both treatment groups. The incidence and severity of lymphoplasmacytic perivascular cuffing, pulmonary congestion, and pulmonary edema were similar in both groups. Additionally, one cat in the T02 vaccinated group had minimal alveolar thrombi. Overall, there were no substantial differences in microscopic pathology in nasal turbinates, trachea, bronchi, and lungs between the two treatment groups.

## 4. Discussion

Since the start of the SARS-CoV-2 pandemic, multiple animal species were found to be highly susceptible to SARS-CoV-2 infection, including hamsters, white-tailed deer, ferrets, mink, domestic and wild felids, and others [2,3,4,5,6,7,8,9,10,11,12,13,14,15]. Multiple authors, including ourselves, have demonstrated that cats are both highly susceptible to infection caused by SARS-CoV-2 and can transmit the virus to sentinel animals with high efficiency [31,32,33,34,41]. Cats are ubiquitous around the world; over 45 million USA households own at least one cat and roughly 50 to 70 million feral cats are estimated to be present in the USA alone. Therefore, there is concern that cats can play an active role in the maintenance and spread of SARS-CoV-2. Therefore, the development of a SARS-CoV-2 vaccine for cats is important in order to protect humans from zoonotic SARS-CoV-2 infections.

The objective of this work was to evaluate the safety and efficacy of a SARS-CoV-2 recombinant SARS-CoV-2 spike protein subunit vaccine developed by Zoetis. In the experimental trial, one group (group T02) of four cats was immunized with two doses of the experimental SARS-CoV-2 spike protein-based vaccine, and another group of four cats (group T01) was treated with placebo. Immunizations were administered at a three-week interval. All cats vaccinated with the SARS-CoV-2 spike protein seroconverted and had high SARS-CoV-2-specific ELISA and neutralizing antibody titers at the time of challenge seven weeks after booster vaccination (Table 2 and Table 3). Cats were challenged simultaneously by the intranasal and oral route with the Wuhan-like SARS-CoV-2 strain USA-WA1/2020. The vaccinated cats mounted a strong anamnestic response 2 weeks after SARS-CoV-2 infection (Table 2 and Table 3). Differences in viral RNA shedding between the two groups from different cavities (nasal, oral, rectal) were the primary parameters for determination of vaccine efficacy. Serologic responses, macroscopic and microscopic lesions, as well as clinical signs in vaccinated animals (group T02) as compared to control animals (group T01) were considered supporting variables.

Post-vaccination, animals of the T02 vaccinated group showed robust immune responses to vaccination as demonstrated by high titers of neutralizing antibodies and ELISA antibodies at the time of challenge (day 0). At the same time point (day 0), the mock-vaccinated animals of the T01 placebo control group remained seronegative, thus confirming the validity of the vaccination-challenge experiment. On day 14 post SARS-CoV-2 challenge (), a significant anamnestic immune response with both neutralizing and ELISA antibody titers was observed in vaccinated animals, but not in control animals (*p* < 0.0001 on days 0 and 14 post-challenge). This demonstrates that the SARS-CoV-2 spike protein-based vaccine induces strong serologic responses post-vaccination as well as significantly increased antibody responses in vaccinates post-challenge, providing a strong indication for the efficacy of the vaccine.

RT-qPCR testing for the presence of viral RNA in nasal and oral swabs demonstrated a significant reduction in viral shedding in T02 vaccinates compared to the T01 control animals. A significant difference was observed between the T02 vaccinates and the T01 controls in nasal shedding for both, the number of viral RNA copies, measured as the area under the curve (AUC), and the duration of shedding (*p* = 0.01274 and *p* = 0.0060; Table 3). Similarly, a significant difference was observed between the T02 vaccinates and the T01 controls in oral shedding for both, the AUC and the duration of shedding (*p* = 0.01471 and *p* = 0.0587, respectively; Table 3). For rectal shedding, a reduction in virus shedding was observed in T02 vaccinates, but this was statistically significant only on day 2 (*p* < 0.0001) post-challenge. A difference was observed between the T02 vaccinates and the T01 controls in rectal shedding for the duration of shedding, but this was statistically not significant (*p* = 0.0547). Overall, the vaccine was able to significantly decrease the amount and duration of oral and nasal shedding and is considered highly efficacious in vaccinated animals challenged with a virulent SARS-CoV-2 strain.

Vaccination of cats with the SARS-CoV-2 recombinant spike protein vaccine did not result in any significant side effects or clinical signs, indicating that the vaccine is safe. The challenge with the Wuhan-like SARS-CoV-2 strain did not elicit any clinical signs (e.g., conjunctivitis, depression, dehydration, nasal congestion, nasal discharge, cough, ocular discharge, sneezing, death, or other) in either the control or vaccinated animals, except for fever in one control animal of the T01 placebo control group which experienced episodic light fever starting on day 2 and ending on day 10 (ranging from 103.2 °F to 103.5 °F). The lack of clinical findings is consistent with our previous findings in similar-age cats experimentally challenged with the identical SARS-CoV-2 USA-WA1/2020 strain [32,41]; in these previous studies, all infected animals were clinically asymptomatic throughout the entire post-challenge observation period [32,41]. These results are also in agreement with other published data, where intranasal/per os experimental infection of juvenile/adult cats was found to be asymptomatic or resulted only in mild respiratory disease [31,34,42].

Necropsy on day 14 post-SARS-CoV-2 challenge revealed macroscopic changes such as congestion, discoloration, and atelectasis in the lungs of animals in both treatment groups. All observed gross lesions were relatively mild with no substantial differences between the two treatment groups. Microscopic lesions, observed in the lungs, trachea, bronchi, and nasal turbinates, were mostly mild to moderate and were similar in both treatment groups (Table 4, Figure 1). These data provide another measure for the safety of the subunit COVID-19 vaccine, as there was no enhancement in lesion incidence or severity in vaccinated animals compared to controls. Since necropsy took place 14 days after challenge, it is assumed that any challenge-induced acute lesions were mostly resolved in both treatment groups. The low incidence and severity of microscopic lesions correlated with the clinical data, where virtually no clinical signs were observed in the majority of animals enrolled in the study; and with the fact that, at 14 days after challenge, viral RNA was found only in the trachea of one animal in the T01 control group but not at all in any tissues of the T02 vaccinated animals.

For virus challenge, the prototype Wuhan-like SARS-CoV-2 USA-WA1/2020 strain was used as the challenge virus. Due to the rapid molecular evolution of SARS-CoV-2, new virus variants of concern (VOCs) continue to emerge, and they have been able to break through immune responses mounted against previously circulating strains. Several human vaccines were recently upgraded to include new SARS-CoV-2 VOCs to ensure adequate protection following vaccination. Therefore, in a separate study, the potential cross-protection of the Zoetis spike-based subunit vaccine was evaluated against the SARS-CoV-2 Delta VOC (B.1.617.2). Notably, serum from cats vaccinated with the Zoetis spike subunit vaccine was able to neutralize the SARS-CoV-2 Delta VOC at comparable levels to the Wuhan-like SARS-CoV-2 strain [43]. This suggests that the subunit Zoetis vaccine is able to protect against multiple SARS-CoV-2 strains including novel VOCs. Additional studies are needed to evaluate the ability of the Zoetis spike subunit vaccine to provide cross-protection against SARS-CoV-2 VOCs currently circulating in humans. In summary, the results of this study demonstrate that the SARS-CoV-2 spike-based subunit vaccine is safe and provides efficient protection against SARS-CoV-2 infection in cats; importantly the vaccine can be easily adapted to new virus variants if deemed necessary.

## 5. Conclusions

A recombinant, subunit vaccine based on the spike protein of SARS-CoV-2 was evaluated in cats. The vaccine significantly decreases both nasal and oral viral RNA shedding as detected using RT-qPCR. Duration of nasal, oral, and rectal viral RNA shedding was also significantly reduced in vaccinates compared to control animals. Our findings support the safety and efficacy of the recombinant SARS-CoV-2 spike protein-based vaccine against SARS-CoV-2 and provide the basis for additional animal studies to facilitate full licensure of the vaccine for cats.

## Figures and Tables

**Table 1:** ELISA antibody titers for each treatment group.

Group	DPC	BT Least Squares Mean	BT Standard Error	BT 90% Confidence Limits	Minimum	Maximum
T01	0	212	62	127–354	150	300
	14	3553	1035	2128–5932	2700	8100
T02	0	42,089 *	12,260	25,211–70,266	24,300	72,900
	14	95,865 *	27,924	57,423–160,043	72,900	218,000

T01, control placebo group; T02, SARS-CoV-2 spike protein vaccine group; DPC, days post-challenge. * Designates significant difference when compared to T01 at the same time point.

**Table 2:** SN antibody titers back-transformed least squares means (LSM) for each treatment group.

Group	DPC	BT Least Squares Mean	BT Standard Error	BT 90% Confidence Limits	Minimum	Maximum
T01	0	10.0	2.49	6.5 to 15.5	10	10
	14	160.0	39.81	103.4 to 247.6	160	160
T02	0	320.0 *	79.61	206.8 to 495.3	160	1280
	14	2560.0 *	636.90	1654.1 to 3962.1	2560 #	2560 #

T01, placebo control group; T02, SARS-CoV-2 spike protein vaccine group; BT, back-transformed; DPC, days post-challenge. * Designates significant difference when compared to T01 at the same time point. # Dilution not end-pointed.

**Table 3:** Nasal, oral, and rectal swab RT-qPCR results by summary of least squares means for each treatment and day.

Swab		Day Post-Challenge							
		0	2	4	6	8	10	12	14
Nasal	T01	0.00 × 10^+00^	2.42 × 10^+09^	2.04 × 10^+10^	1.55 × 10^+09^	5.54 × 10^+07^	4.02 × 10^+05^	2.65 × 10^+05^	1.26 × 10^+05^
	T02	0.00 × 10^+00^	1.64 × 10^+06^ *	2.37 × 10^+05^ *	1.20 × 10^+06^ *	8.36 × 10^+03^ *	0.00 × 10^+00^ *	1.41 × 10^+01^ *	0.00 × 10^+00^ *
Oral	T01	0.00 × 10^+00^	9.71 × 10^+08^	9.64 × 10^+08^	2.12 × 10^+08^	1.84 × 10^+04^	1.49 × 10^+01^	1.15 × 10^+03^	2.60 × 10^+02^
	T02	0.00 × 10^+00^	6.00 × 10^+04^ *	3.66 × 10^+07^	5.18 × 10^+06^	1.95 × 10^+01^ *	1.55 × 10^+01^	2.95 × 10^+02^	0.00 × 10^+00^ *
Rectal	T01	0.00 × 10^+00^	5.81 × 10^+08^	1.08 × 10^+08^	1.48 × 10^+10^	1.90 × 10^+07^	1.16 × 10^+06^	1.26 × 10^+05^	4.83 × 10^+03^
	T02	0.00 × 10^+00^	0.00 × 10^+00^ *	5.52 × 10^+05^	3.74 × 10^+09^	1.72 × 10^+08^	2.92 × 10^+04^	2.50 × 10^+02^	1.57 × 10^+01^

* Designates significant difference when compared to T01 at the same time point.

**Table 4:** Individual and group average histopathology severity scores.

		Nasal Turbinate			Trachea/Bronchi			Lungs						
Group	Cat ID	Epithelial Changes	Gland/Duct Changes	Inflammation	Epithelial Changes	Gland/Duct Changes	Inflammation	Alveolar space, Septal Changes	Bronchi/Intraluminal Exudate	Perivascular Cuffing	Congestion	Edema	Bronchi/Bronchiole Epithelial	Focal Alveolar Thrombi
T01	M194474	2	1	3	1	1	0	0	2	0	3.5	2	0	0
T01	M192790	2	1	4	2	2	0	1	2.5	2	3.5	2	0	0
T01	M192144	2	1	1.5	1	2	0	1.5	1.5	1.5	4	2	0	0
T01	M191539	0	1.5	2	0	2	0	0	1	0	3	2	0	0
	AVG (SD)	1.5 (0.93)	1.1 (0.35)	2.6 (1.06)	1.0 (0.76)	1.75 (0.46)	0 (0)	0.63 (0.74)	1.75 (0.7)	0.88 (0.99)	3.5 (0.53)	2.0 (0)	0 (0)	0 (0)
T02	M194229	0	3	2.5	1	1	0	1	1	0	3.5	2	0	0
T02	M192812	2	1	1.5	1	3	1	2	1	2	3.5	2	0	1
T02	M192128	0	1.5	2.5	0	1	0	1	1.5	1	3.5	2	0	0
T02	M191806	2	1	3.5	0	1	0	1.5	1.5	1	3	2	0	0
	AVG (SD)	1.0 (1.07)	1.2 (0.92)	2.5 (0.93)	0.5 (0.86)	1.5 (0.93)	0.25 (0.46)	1.4 (0.52)	1.13 (0.46)	1 (0.75)	3.38 (0.52)	2.0 (0)	0 (0)	0.25 (0.46)

Average severity scores determined by two independent veterinary pathologists are shown as group average (AVG) and standard deviation (SD).

## Data Availability

Data is available upon request.
